# The Underutilization of National Diabetes Prevention Program Among Women With a History of Gestational Diabetes Mellitus: An In-Depth Focus Group Analysis

**DOI:** 10.1016/j.focus.2024.100279

**Published:** 2024-10-05

**Authors:** Priyadharshini Narayanan, Sahitya R. Kothapalli, Harmonie B. Strohl, Linda L. Hill

**Affiliations:** 1 UC San Diego School of Medicine, San Diego, California; 2 Independent Researcher; 3 Herbert Wertheim School of Public Health and Human Longevity Science, UC San Diego, San Diego, California

**Keywords:** Gestational diabetes, National Diabetes Prevention Program, NDPP participation, diabetes prevention

## Abstract

**Introduction:**

Gestational diabetes mellitus significantly increases the risk of developing Type 2 diabetes mellitus in women. However, the National Diabetes Prevention Program, effective in reducing Type 2 diabetes mellitus incidence, is underutilized among women with a history of gestational diabetes mellitus. This study aims to explore the factors contributing to this underutilization and propose strategies to increase National Diabetes Prevention Program's usage.

**Methods:**

A qualitative study with narrative methodology was employed, involving focus group discussions with 18 women aged between 29 and 43 years, who had a history of gestational diabetes mellitus. Participants were recruited from a gestational diabetes mellitus registry through University of California, San Diego's online platform. The discussions were aimed at understanding their awareness of Type 2 diabetes mellitus risk after gestational diabetes mellitus and their knowledge about the National Diabetes Prevention Program.

**Results:**

Thematic analysis of the discussions revealed 4 key themes: (1) challenges in navigating health information after gestational diabetes mellitus; (2) perceived facilitators for National Diabetes Prevention Program participation, including peer support, personalized guidance, and virtual options; (3) perceived barriers, such as time constraints, childcare responsibilities, and financial considerations; and (4) insights into patient perspectives on gestational diabetes mellitus care. Despite awareness of their increased Type 2 diabetes mellitus risk, there was a notable gap in knowledge about the National Diabetes Prevention Program among participants.

**Conclusions:**

The study highlights the critical need for improved communication regarding postgestational diabetes mellitus risk of type 2 diabetes and the National Diabetes Prevention Program. Addressing communication gaps, offering personalized support, and accommodating the unique challenges faced by women with a history of gestational diabetes mellitus can enhance National Diabetes Prevention Program participation. The significance of a patient-centric approach in diabetes prevention is underscored, emphasizing that such an approach could significantly impact Type 2 diabetes mellitus prevention in this high-risk population.

## HIGHLIGHTS

• The National Diabetes Prevention Program (NDPP) is an underutilized resource among women who have had gestational diabetes.
• These women need better health communication about the NDPP.
• Women are interested but not confident about enrolling in the program.
• Experiences with health care during pregnancy may impact decisions to enroll in NDPP.
• Childcare and time constraints are barriers to enrollment in NDPP.

## Graphical abstract

**Figure:**
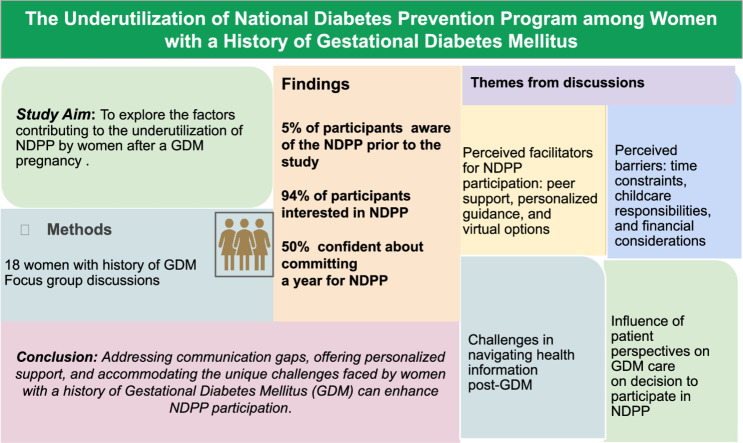

## INTRODUCTION

Gestational diabetes mellitus (GDM) is defined as the first diagnosis of glucose intolerance during pregnancy.^1^ It is estimated that 2%–10% of pregnancies in the U.S. are affected by gestational diabetes every year.^1^ This rate has been increasing in the past decade.^2^ Although GDM resolves with delivery of child, it is a harbinger of future Type 2 diabetes mellitus (T2DM).^3^ The odds of developing T2DM in the decade after GDM are 6–17 times the odds with no history of GDM.^3^, ^4^, ^5^ There is sufficient evidence that this consequence of GDM is largely preventable. The landmark Diabetes Prevention Program trial demonstrated that lifestyle modifications targeting 7%–10% reduction in weight can reduce diabetes incidence by 50% among women with previous GDM.^3^ The Centers for Disease Control and Prevention's National Diabetes Prevention Program (NDPP) was developed on the basis of this evidence and has been in effect for 13 years.^4^

The NDPP is recommended for individuals at risk for T2DM, including women with a history of GDM.^4^ Various outcome studies and evaluation of NDPP have shown that it tends to reach primarily older adults who are aged 56.8 years on average.^6^, ^7^ A study evaluating the reach of NDPP for young women found that women of any age with past gestational diabetes were less likely to enroll than women with other risk factors for diabetes (OR=0.44; 95% CI=0.35, 0.55), suggesting that the NDPP may be an underutilized resource for this high-risk population.^8^ It is unclear why women with history of GDM form a low proportion of NDPP enrollees. To address this gap, this study sought to understand the reasons through a focus group discussion involving women with a history of GDM.

Thus, the aim of this study was to explore women's perspectives on the feasibility of participating in the NDPP within 1–3 years after delivery of a GDM pregnancy, in addition to gleaning insights from their experiences with GDM. Furthermore, findings from this study would be useful in promoting increased participation of women with a history of GDM in the NDPP.

## METHODS

In terms of author positionality, there are 4 authors for this study. Two are Indian Americans, and the other 2 are White Americans. The primary author (PN), who led the data collection and analysis process, has experience in primary care and preventive medicine. Data extraction and analysis were performed by coauthor SRK who has experience in public health but no background in clinical medicine. This arrangement was chosen with the intention to minimize any cultural or theoretical influence on the study.

### Study Sample

Qualitative research with a narrative approach was utilized for this study relying on focus group discussions.^9^ This focus group study was part of a quality improvement project implemented at University of California, San Diego (UCSD) Health to facilitate access to the NDPP for women with history of GDM. Approval was obtained from the UCSD Aligning and Coordinating Quality Improvement, Research, and Evaluation committee. The focus group discussions were guided by the principal investigator (PN) with the assistance from a research team member (HS). The principal investigator (PN) has expertise in primary care and preventive medicine. The researchers did not have any previous relations with the study participants. Participants were informed prior to each session that the discussion would be recorded and remain confidential. Informed written consent was obtained from all participants, and they were compensated for their time with a $25 visa gift card.

### Measures

Potential participants were identified through a GDM registry and recruited by an electronic letter disseminated through UCSD's online information platform (MyChart). Women aged >18 years with history of GDM who delivered at a UCSD facility or currently pregnant with GDM and receiving prepartum care at UCSD were included in the study. Letters were sent to 300 eligible women. Focus group sessions were conducted through the virtual platform Zoom for all participants. Five virtual meetings of 2–5 individuals were held between May and July 2023. No repeat interviews were conducted. All focus groups were conducted in English with an average time of 60 minutes per focus group. The sessions utilized open-ended questions designed to elicit information about women's awareness and understanding of their future risk of T2DM, the existence of NDPP, and their perspectives on ability to participate in the NDPP (Table 1). If any single participant within a group answered *No* to Question 2 (Table 1), the focus group was paused, and a 5-minute information session on NDPP was presented orally by the author (PN) along with the use of a PowerPoint slide (transcript of the presentation is provided in Appendix, available online).

**Table 1:** Open-Ended Guided Questions for Participants

Questions
1. What is your understanding of what happens to your health after a gestational diabetes pregnancy?
2. Have you heard of the Center for Disease Control and Prevention's National Diabetes Prevention Program?
3. After hearing about the National Diabetes Prevention Program, if given the opportunity would you be interested in enrolling and committing to a program in the next 6 months–2 years?
4. If answered yes to question 3, why yes and what makes you confident that you can commit to the program? If answered no to question 3, why not?
5. If not due to time/childcare barriers, do you think having an app- based, self-paced Diabetes prevention program led by coach would work better for you?

### Data Analysis

A thematic analysis approach was used to analyze gathered data.^10^ Data analysis was conducted by 2 members of the research team (PN and SRK). The audio files from the focus groups were transcribed using Go Transcribe transcription service. These transcriptions were verified for accuracy against the original audio files independently by 2 members of the research team. Next, each line of transcript was reviewed by each member, who manually identified and highlighted key points. This process involved identifying the repetitions and points of agreement among participants, which were then grouped into initial themes. Highlighted transcriptions from each focus group were reviewed several times; notes were taken to capture reasoning behind key points and observations. These reasons provided additional context and depth to identified themes. After this independent analysis, the researchers met to discuss the findings, and any discrepancies in categorizing the themes and subthemes and/or interpreting the data were resolved through multiple collaborative discussions among the 2 research team members. Subsequently**,** team members refined their analyses independently on the basis of discussions. Final themes and subthemes were then reviewed by a third reviewer (LH), who provided additional perspectives on the interpretation. This repeated process led to the final framework that described and assessed the information and views conveyed by the participants.

## RESULTS

All 18 participants completed a questionnaire providing demographic information, including age, race and ethnicity, the number of pregnancies with GDM, current pregnancy or postpregnancy status, educational background, employment status, and the type of health insurance (Table 2). The mean maternal age in this study was 36.83 years. Most of the participants were White (*n*=8, 44.44%) and had a postgraduate education (*n*=11, 61.11%). Two thirds of the participants were multiparous, and 80% of the participants had GDM during their first pregnancy. A high proportion of the women had preferred provider organization health insurance with low deductibles. Majority of participants were from northern areas of San Diego, especially concentrated around UCSD. Most participants were working or planning to join work soon. About 20% of the participants were prescribed metformin on the basis of medical chart review. None of the participants had previously participated in an NDPP.

**Table 2:** Characteristics of the Participants Across 5 GDM Focus Groups

Characteristic	Total (N=18)	
	Total	%
Race		
White	8	44.44
African American	1	5.55
Asian	2	11.11
South Asian	2	11.11
Middle Eastern	3	16.66
Unknown	2	11.11
Ethnicity		
Hispanic	2	11.11
Non-Hispanic	14	77.78
Unknown	2	11.11
Education		
High school	1	5.55
College	4	22.22
Postgraduate	11	61.11
Unknown	2	11.11
Health insurance		
HMO	5	27.77
PPO	10	55.55
Medi-Cal	1	5.55
Unknown	2	11.11
Deductible		
$0	7	38.88
≤$500	3	16.66
>$500	4	22.22
Do not know	4	22.22
Copay		
$0	1	5.55
<$15 per office visit	4	22.22
>$15 per office visit	8	44.44
Do not know	5	27.77
Total number of pregnancies		
1	6	33.33
>1	12	66.67
No of pregnancies with GDM		
1	16	88.89
>1	2	11.11
Working status		
Currently working or scheduled to return to work	15	83.33
Not currently working/scheduled to return to work	3	16.67
Age^a^	36.83	4.16
San Diego County geographic location		
North Coastal	2	11.11
North Inland	3	16.66
North Central	8	44.44
East	0	0
Central	1	5.55
South	2	11.11
Another county	2	11.11
Prescribed metformin	3	20

a Continuous variable, *mean and standard deviation*. GDM, gestational diabetes mellitus; HMO, health maintenance organization; PPO, preferred provider organization.

Table 3 summarizes participants' interest and confidence for commitment to NDPP across the 5 focus groups. All participants (*N*=18) demonstrated heightened awareness of their elevated risk for Type 2 diabetes, despite limited prior knowledge of the Center for Disease Control and Prevention's NDPP. Among the 5 focus groups, varying degrees of interest and confidence to commit to the NDPP were observed, with some groups expressing confidence in commitment to NDPP, whereas others showed mixed feelings. Overall, 94.4% of all participants expressed a significant interest in engaging in the NDPP, but only 50% were confident about their ability to commit to the program.

**Table 3:** Summary of Participant's Interest and Confidence to Commit to NDPP Across the 5 GDM Focus Groups

Focus group	Total participants (N)	Interest to participate, *n* (%)	Confidence in committing, *n* (%)
Group 1	5	5 (100)	4 (80)
Group 2	3	3 (100)	1 (33)
Group 3	3	2 (67)	1 (33)
Group 4	2	2 (100)	1 (50)
Group 5	5	5 (100)	3 (60)
Total	N=18	*n*=17 (94.4%)	*n*=10 (50%)

GDM, gestational diabetes mellitus; NDPP, National Diabetes Prevention Program.

These results shed light on the need to further analyze the responses regarding barriers to the program commitment among women with a history of GDM. The qualitative findings derived from the focus group discussions highlight a diverse range of perspectives among the total participants, which were discussed in the thematic analysis.

### Thematic Analysis

Through an in-depth examination of participant's accounts, several key themes emerged, shedding light on the complexities and challenges individuals face in the aftermath of GDM. The emerging themes encompass navigating preventive health care after GDM pregnancy, perceived barriers and facilitators for NDPP participation and commitment, and patient experiences during their GDM care that influence their healthcare decisions. These themes collectively provide a comprehensive view of the complexities individuals encountered after GDM, informing potential interventions for healthcare providers (HCPs).

### Theme 1: Navigating Health Information After Gestational Diabetes Mellitus

The theme revolves around the knowledge and awareness individuals have regarding the risks of developing diabetes mellitus after pregnancy, with a specific focus on gaps in navigating health information in the post-GDM period.

**Subtheme 1.1: Knowledge and awareness.** General awareness and knowledge about increased risk of developing diabetes mellitus in the future was excellent. However, some women commented on the lack of information on the extent of this risk. In contrast, awareness of the existence of the NDPP was minimal among participants.

Participant quotes include the following: “I understand that you are at a higher risk of developing type 2 diabetes” (G5P1), “I also, from what I understand your baby has a lifelong higher risk of developing type 2 diabetes and that after I've already been through this, but after you do your A1Cs pretty regularly with your PCP” (G5P2), and “I was through the diabetes pregnancy program, you know, pamphlets after pamphlets and sheets and this and that, but like, I didn't, I don't remember seeing this (NDPP)” (G5P2).

**Subtheme 1.2: Communication gaps in advice after delivery.** Participants reflected on the postdelivery health advice and education about preventive measures to mitigate the risk of developing T2DM after experiencing GDM. Some participants expressed a sense of inadequate information provided by HCPs in managing diabetes mellitus after delivery. Few participants relied on personal research conducted through the internet to fill in the gaps of information. This suggests the need for clear and more consistent guidance to bridge the communication gaps after GDM.

Participant quotes include the following: “Yes, I was told there is higher risk of future type 2 diabetes, but I wasn't given a percentage, like how many women out of how many women typically develop it but yes I was told there is risk” (G5P3), “Mine was the same, I was told there is risk, but I wasn't given any further information on how high the risk was” (G5P4), and “I'm very nervous too. Okay, right now, following the diet, but once the baby is here, am I supposed to be eating the same way? Nobody has told me that” (G1P2).

### Theme 2: Perceived Facilitators for Participation in National Diabetes Prevention Program

This theme digs deeper into the motivational aspects influencing enrollment in NDPP after GDM. Most of the participants resonate in alignment with peer support, personalized guidance, and a desire for a healthy lifestyle as primary motivators. Participants shared the pivotal role of peer support in their decision to join NDPP. Shared experiences and encouragement from others who have faced similar challenges create a sense of community, becoming a powerful motivator for sustained commitment.^11^, ^12^ The significance of in-person sessions and face-to-face guidance is evident in participants' accounts, emphasizing that the personalized nature of these interactions contributes to a more meaningful experience. Finally, personal commitment emerged as a potent driving force for some participants to prevent T2DM.

Participant quotes include the following: “For me, to have a group that you are checking in with that is going through a similar experience…I just think to me it would be motivating to be in group” (G1P2), “For me, it's just my health overall. I mean, I want to do everything I can to prevent getting the type 2 diabetes” (G5P2), and “It's hard...it's hard to eat healthy, it's hard to exercise, to find the time for it and when you are in a group other people can motivate you but other people can also give you ideas and that's really important” (G5P4).

### Theme 3: Perceived Barriers to Participation in National Diabetes Prevention Program

This theme uncovers the barriers affecting participants' enrollment in the NDPP after GDM, revealing factors influencing their commitment. Key issues include accessibility, financial considerations, lack of postpartum support, and balancing work and childcare, all limiting participation. Some individuals expressed confidence in managing diabetes mellitus independently, emphasizing the need for tailored approaches. Motherhood presented unique challenges, with childcare responsibilities and professional demands as potential barriers to NDPP enrollment. Participants stressed the need for tailored support, flexibility, and accommodating schedules. In addition, health insurance coverage and the availability of a virtual delivery option are critical considerations impacting decisions. Participants recognized the practicality of virtual formats to accommodate their diverse schedules, increasing the program's reach.

Participant quotes include the following: “I think if I would join it might later down the line like when the second baby is already sent to school/daycare. Cuz I'm a full-time working mother as well. And so, if it's during my working time, I probably can just squeeze in a little bit of time” (G1P4), “I would like to. I'd be interested in it, but realistically, I don't think I would be able to commit. So once... I think once a week for the first six months, it'd be the hardest part just because of work and I have two kids and my life is chaos” (G4P2), “I would be interested. I would just have a lot of barriers, I guess, like childcare would be a big issue for me. My work hours are kind of wonky too, but I mean, if I could, if I could get it, I would work with my schedule and not have a childcare issue, I would do it” (G5P4), “And then also we are both self-employed and then we pay..Yeah, insurance out of our own pocket. So that's another issue.” (G3P2), “I'm self-employed. So I don't have sick days. I don't have benefits” (G4P4), and “the money again, the same thing with like how much would insurance cover, if I'm already going to be paying for my own insurance, my son's insurance and then just another expense out of pocket” (G4P3).

### Theme 4: Impact of Patient–Provider Relationships From Previous Pregnancies

Throughout multiple sessions, the significant impact of HCPs on patients emerged as a recurring theme. Women shared their experiences with primary care physicians, midwives, dieticians, and nurses during their GDM pregnancies. These experiences influenced their perceptions, whereas positive interactions reinforced trust in NDPP, and negative ones fostered skepticism. Some participants expressed anxiety and stress associated with GDM, revealing emotional challenges that may affect adherence to postdelivery preventive care recommendations. Participants frustration with conflicting guidance from different HCPs underscored the importance of clear and consistent communication to maintain patient trust. In addition, one participant experienced GDM-related stigmatization, and another shared the deficiency of cultural understanding and accommodations in existing nutritional programs.

Participant quotes include the following: “Cuz (during pregnancy) I got so much information and really empowered me with a lot of stuff, and I had nutritionist that I could call and email and be in contact with” (G1P1), “I mean, I was... I know when I was pregnant, they were checking on me every week, that actually helped me keep on track with obtaining my goals because they help us set those smart goals” (G1P2), “I am upset at the situation because knowing my high-risk and knowing all the information that they gave me, I felt like it was like, okay we're taking care of you while you have the baby but then after you have a baby then you know, we don't really care about you anymore” (G3P1), “It's just so frustrating how conflicting information out there is... when I was with the midwives and even my internal medicine and the dietary professional after delivery, everyone kept brushing it off, like it's no big deal and it's just going to be one A1c test while I am seen at my annual physical” (G3P2), and “I felt like I couldn't have access to my home foods during... like what I needed to be comfortable during my pregnancy. I couldn't eat those things because even the meal plan and everything was like chicken and broccoli… I don't eat chicken and broccoli, but okay, I'll force myself to eat these things” (G5P2).

## DISCUSSION

This study provided valuable information from the focus group sessions on participants' perspectives on enrolling in the NDDP within 1–3 years after delivery of GDM pregnancy. These findings provided a better understanding of challenges and opportunities experienced by women to prevent T2DM. Most of the participants exhibited heightened awareness of the future risk of T2DM, suggesting that the existing programs aimed at educating women about the risks associated with gestational diabetes are successful. However, the study revealed deficiencies in health education and communication about the Centers for Disease Control and Prevention's NDPP itself for women with history of GDM. Although the researchers hypothesized that time constraints and competing priorities would be the major barrier to enrollment, this study brought to light that the most significant barrier may be the lack of information on NDPP. About 50% of the study participants were willing to commit to the yearlong program, demonstrating that women are highly self-motivated and ready to utilize available resources to prevent diabetes. This suggests the need to improve the dissemination of information after pregnancy. Awareness about the NDPP and potential participation could be improved by providing information and handouts on NDPP during a GDM pregnancy toward the end of pregnancy and at the time of discharge after delivery by diabetes health educators and obstetricians. This could also provide a head start for women to plan and find a program that is logistically convenient for them. Electronic medical records systems could be used to provide automated triggers to place an NDPP referral for a diagnosis of history of gestational diabetes on chart. Many participants noted that peer support and personal guidance were compelling motivational factors about NDPP. Promoting peer support through exclusive NDPP cohorts of women with a history of GDM and connecting new mothers with other trained peer facilitators to foster positive group dynamic may improve long-term commitment in this study population. The study findings also revealed that managing blood sugars during GDM was very stressful for some participants because the dietary education conformed to typical American food. Modifications and expansions of diabetes prevention programs with dietary information that is culturally inclusive are required to address this gap. Finally, the study highlights financial and logistical barriers among the study population, including the challenges faced by self-employed women and childcare responsibilities, which are essential to consider and address to help women participate in and complete the NDPP.

This study had several strengths. It is the first qualitative study, to our knowledge, exploring barriers and facilitators for NDPP enrollment among women with a history of GDM. This study had a good diversity of racial and ethnic representation, including immigrant women, increasing its external validity. Robust participation in the discussions by individuals, along with use of thorough analytical methods, contributes to credibility of the results.

### Limitations

However, this study had some limitations. First, it is limited by the small sample size, which may limit the generalizability of results. This study had a higher proportion of participants with college and higher education, with private insurance, and living in areas of San Diego considered affluent and thus potentially reflect challenges to enrollment in NDPP faced by women who are of moderate-to-high socioeconomic backgrounds. Future studies are recommended to evaluate challenges faced by women of different socioeconomic backgrounds and to evaluate whether the findings of this study are replicated in other parts of the U.S. In addition, studies could investigate how variations in cultural factors impact the participation in NDPP. This would inform public health departments as well as healthcare institutions to develop strategies toward increasing NDPP enrollment tailored to diverse populations. In 2 groups, the discussion was influenced by highly vocal participants, which may hinder capturing certain viewpoints. There is some potential for social desirability bias given the sensitive nature of the topic, especially on healthcare utilization at personal level.

## CONCLUSIONS

Despite the existence of resources such as NDPP, there are deficiencies in healthcare information and education that perhaps contribute to poor enrollments of women with history of GDM in NDPP. It is essential to take immediate action to widely disseminate information on NDPP to this high-risk population. In addition, HCPs focus on patient-centric approach in GDM care, emphasizing that trust and communication after delivery benefit this study population.

## Appendix Supplementary materials

Image, application 1

## Footnotes

Supplementary material associated with this article can be found in the online version at doi:10.1016/j.focus.2024.100279.

## References

[1] Gestational diabetes. Centers for disease control and prevention. https://www.cdc.gov/diabetes/basics/gestational.html. Updated May 15, 2024. Accessed November 6, 2023.

[2] Gregory CWE, Danielle ME. (July 19, 2022).

[3] Kim C, Newton KM, Knopp RH. (2002). Gestational Diabetes and the Incidence of type 2 diabetes: a systematic review. Diabetes Care.

[4] Dennison RA, Chen ES, Green ME (2021). The absolute and relative risk of type 2 diabetes after gestational diabetes: a systematic review and meta-analysis of 129 studies. Diabetes Res Clin Pract.

[5] Juan J, Sun Y, Wei Y, et al. Progression to type 2 diabetes mellitus after gestational diabetes mellitus diagnosed by IADPSG criteria: systematic review and meta-analysis. *Front Endocrinol*. 2022;13:1012244. 10.3389/fendo.2022.1012244. PMC9582268 36277725

[6] Ritchie ND, Seely EW, Nicklas JM, Levkoff SE. (2023). Effectiveness of the National diabetes prevention program after gestational diabetes. Am J Prev Med.

[7] Sauder KA, Ritchie ND, Crowe B, Cox E, Hudson M, Wadhwa S. (2021). Participation and weight loss in online National Diabetes Prevention Programs: a comparison of age and gender subgroups. Transl Behav Med.

[8] Ritchie ND, Sauder KA, Fabbri S. (2017). Reach and effectiveness of the National diabetes prevention program for young women. Am J Prev Med.

[9] Kitzinger J. (1995). Qualitative research. Introducing focus groups. BMJ.

[10] Silverman D. (2011). https://www.researchgate.net/publication/261554924_Interpreting_Qualitative_Data_A_Guide_to_the_Principles_of_Qualitative_Research.

[11] Ingstrup MS, Wozniak LA, Mathe N (2019). Women's experience with peer counselling and social support during a lifestyle intervention among women with a previous gestational diabetes pregnancy. Health Psychol Behav Med.

[12] Azmiardi A, Murti B, Febrinasari RP, Tamtomo DG. (2021). The effect of peer support in diabetes self-management education on glycemic control in patients with type 2 diabetes: a systematic review and meta-analysis. Epidemiol Health.

