# Supplementary material for: The Underutilization of National Diabetes Prevention Program Among Women With a History of Gestational Diabetes Mellitus: An In-Depth Focus Group Analysis
Source: AJPM Focus. 2024 Oct 5;4(1):100279. doi: 10.1016/j.focus.2024.100279 (PMC11994036; doi:10.1016/j.focus.2024.100279)
Supplement: Supplementary file 1 [file mmc1.docx]

**APPENDIX**

**Brief introduction to NDPP provided to participants during focus group discussions.**

‘The National Diabetes Prevention Program is a program that was developed by the Centers for Disease Prevention and Control (CDC). This CDC’s program is run by various organizations in the country like UCSD, Sharp Steely and Scripps Medical. The participants have a risk of future diabetes, whether it's because of prediabetes or because of the history of gestational diabetes or other risk factors for diabetes and all these people are eligible to participate. It is a one-year program. In the first six months of the program, participants meet once every week in a group session in person or online just like this one. While it is going to be group sessions, it is led by a lifestyle coach…And they help you to make better lifestyle choices. The meetings are once a week for the first few months and then later, by the ninth month, it is once a month.

They(coaches) guide you on lifestyle changes. So having, you know, having to go to work, having to take care of the family and still finding time to do your exercises and making good dietary choices, when you go grocery shopping how to read the labels, learn how to get back on track when there is relapse. A very large study was conducted with thousands of people who have pre-diabetes and what they found was when there is one year of intensive lifestyle changes with the help of a coach in a group setting and you are not just learning, but you're also learning to be accountable to other people for the next week and then you achieving your goals, the risk of type 2 diabetes was reduced by 58% in the following 10years. The program is covered by most health insurances including Medicaid. San Diego organizations that are providing diabetes prevention program. There's Scripps medical, skinny jean, Family Health Centers of San Diego, San Yisidro medical. And there are other east coast based online programs that maybe helpful for people who prefer early morning appointment with the east coast timing and there is app based self-paced DPP where you have a lifestyle coach and online community but no group sessions’.
